# Bivalent Genes Targeting of Glioma Heterogeneity and Plasticity

**DOI:** 10.3390/ijms22020540

**Published:** 2021-01-07

**Authors:** Mariam Markouli, Dimitrios Strepkos, Kostas A. Papavassiliou, Athanasios G. Papavassiliou, Christina Piperi

**Affiliations:** Department of Biological Chemistry, Medical School, National and Kapodistrian University of Athens, 11527 Athens, Greece; myriam.markouli@gmail.com (M.M.); smd1700150@uoa.gr (D.S.); kpapavassiliou@gmail.com (K.A.P.)

**Keywords:** bivalency, bivalent genes, histones, glioma, glioblastoma, cancer, HOX genes, epigenetics, glioma therapy

## Abstract

Gliomas account for most primary Central Nervous System (CNS) neoplasms, characterized by high aggressiveness and low survival rates. Despite the immense research efforts, there is a small improvement in glioma survival rates, mostly attributed to their heterogeneity and complex pathophysiology. Recent data indicate the delicate interplay of genetic and epigenetic mechanisms in regulating gene expression and cell differentiation, pointing towards the pivotal role of bivalent genes. Bivalency refers to a property of chromatin to acquire more than one histone marks during the cell cycle and rapidly transition gene expression from an active to a suppressed transcriptional state. Although first identified in embryonal stem cells, bivalent genes have now been associated with tumorigenesis and cancer progression. Emerging evidence indicates the implication of bivalent gene regulation in glioma heterogeneity and plasticity, mainly involving Homeobox genes, Wingless-Type MMTV Integration Site Family Members, Hedgehog protein, and Solute Carrier Family members. These genes control a wide variety of cellular functions, including cellular differentiation during early organism development, regulation of cell growth, invasion, migration, angiogenesis, therapy resistance, and apoptosis. In this review, we discuss the implication of bivalent genes in glioma pathogenesis and their potential therapeutic targeting options.

## 1. Introduction

Gliomas account for the majority of primary central nervous system (CNS) neoplasms, encompassing a diverse set of tumors, commonly of highly aggressive phenotype with limited therapeutic options [1]. The difficulty in treating gliomas mainly stems from their heterogeneity, which makes the response in therapy unpredictable, and, most of the time, underwhelming [2]. Current treatment modalities include the use of radiotherapy, chemotherapy, surgery, and targeted therapy for gliomas. These approaches, however, are largely ineffective, and most tumors recur due to chemo- and radiotherapy resistance [3,4], exhibiting very low five-year-survival rates. In the case of glioblastoma (GBM), the most malignant and aggressive tumor type, less than 5% of patients survive beyond the first five years of diagnosis [5]. The abysmal survival rates and the lack of deeper understanding of glioma pathogenesis and heterogeneity have turned scientific interest in the direction of the study of genetic and epigenetic interplay that regulates gene expression, aiming to identify specific biomarkers and design novel therapeutic approaches.

The genetic profile of gliomas involves several gene mutations associated with their pathogenesis and used for their classification as well as for treatment selection. Gliomas are mainly divided into four grades (World Health Organization (WHO) grade II–IV tumors) based on their histological and molecular features [6]. Most gliomas are characterized by a highly infiltrative growth pattern and are known as ‘diffuse gliomas’ encompassing grade II astrocytomas, grade III anaplastic astrocytomas, and grade IV gliomas (glioblastomas and GBMs). Grade II–IV gliomas are further subgrouped based on the presence of Isocitrate dehydrogenase (IDH) mutations into mutated-IDH and wild type-IDH tumors. Moreover, IDH-mutated GBM may carry epidermal growth factor receptor (EGFR) amplification and chromosome 10 loss, while IDH-wild type GBM harbor hemizygous deletions of the Teneurin Transmembrane Protein 3 (ODZ3). Additionally, GBMs are characterized as epithelioid when they harbor a BRAF substitution of valine to glutamic acid at position 600 (V600E) and often present MYCN amplification. The diagnosis of other glioma subgroups such as Oligodendrogliomas and anaplastic oligodendrogliomas is based on IDH mutation and the 1p/19q codeletion. Moreover, pediatric diffuse high-grade gliomas often harbor mutations in the histone 3 (H3), the substitution of lysine to methionine at the position 37 (K37M) in H3.3 Histone A (H3F3A) gene or less often in Histone cluster 1, H3b (HIST1H3B) gene [6]. Sturm et al. showed that these mutations, combined with key clinical features, such as patient age and tumor location, could help create further subdivisions of glioma subgroups, which are otherwise indistinguishable by histological evaluation [7]. These findings could further favor research within subgroups in order to select specific treatments for each tumor type and improve the design of future clinical trials.

Some of these mutations, like IDH and H3K27M attract epigenetic enzymes and induce epigenetic changes in gliomas, mainly DNA hypermethylation and repressive histone modifications (H3K27me3) that can further control gene expression in a reversible and specific way [8,9,10]. Epigenetic regulation of gene expression has emerged as a pivotal mechanism in glioma progression, occurring through chemical modifications to DNA and histones as well as through non-coding RNAs, micro RNAs (miRNA), and small interfering RNAs (siRNA) that can alter the post-transcriptional fate of gene products [11].

DNA methylation is the most studied mechanism of gene silencing occurring mainly in CpG islands of gene promoters, having been implicated in several diseases, including cancer. However, another level of gene regulation is conferred by the post-translational modifications (PTMs) taking place on histone N-terminal tails, charged with arginine and lysine residues and include methylation, acetylation, phosphorylation, and ubiquitination [12]. These modifications orchestrate the level of chromatin condensation, resulting in the regulation of gene expression within a chromatin segment. Many enzymes, such as histone methylases, demethylases, acetylases, etc., can attach appropriate chemical groups (methyl-, acetyl-, phosphorus, and ubiquitin groups) to histones, thus changing their conformation. In a specific way, methylation, sumoylation, and deacetylation hinder gene transcription by increasing chromatin condensation and making chromatin less accessible to the transcriptional machinery (heterochromatin) [13]. On the contrary, demethylation and acetylation are related to a more “relaxed” chromatin state, involved in increased transcriptional activity (euchromatin) [14].

Histone PTMs are mostly localized in gene promoter as well as gene enhancer regions. They include activating histone marks such as mono-/di-/trimethylation of histone 3 (H3) at lysine 4 (K4), acetylation of H3K27 and H3K9, and trimethylation of H3K36, which promote gene transcription through changes in chromatin structure, making it more available for transcription. On the other hand, repressive epigenetic histone marks such as H3K9me2/3, H3K27me3 promote chromatin condensation and make the binding of the transcription machinery more difficult. The expression of many crucial genes such as *p53* [15], Apolipoprotein E, Homeobox (*Hox*) *A1-7*, *9-11*, *13* [16], and *c-myc* [17], is regulated by these PTMs. Of interest, the co-occurrence of active and repressive histone marks is involved in the transcriptional regulation of bivalent genes associated with the differentiation process in normal embryonic stem cells and cancer cells.

In this review, we discuss general aspects of bivalent chromatin state in the regulation of normal development and differentiation as well as its contribution to the transcriptional plasticity associated with cancer phenotype. We particularly focus on the effect of histone PTMs and bivalent genes in gliomas heterogeneity and plasticity, addressing potential therapeutic targeting options.

## 2. General Aspects of Bivalent Chromatin State

Although bivalency is mainly linked to the pluripotent state of stem cells and gene imprinting, it also represents a global mechanism for tissue-specific gene regulation. Bivalent chromatin domains mainly characterize gene promoters and enhancers, which are decorated with different combinations of histone marks in each specific cell type, depending on their current transcriptional status, thus allowing genes to be switched on or off, based on the receiving signal [18]. These bivalent genes are commonly found in developing organisms even before the onset of zygotic transcription [19].

**Figure 1:**
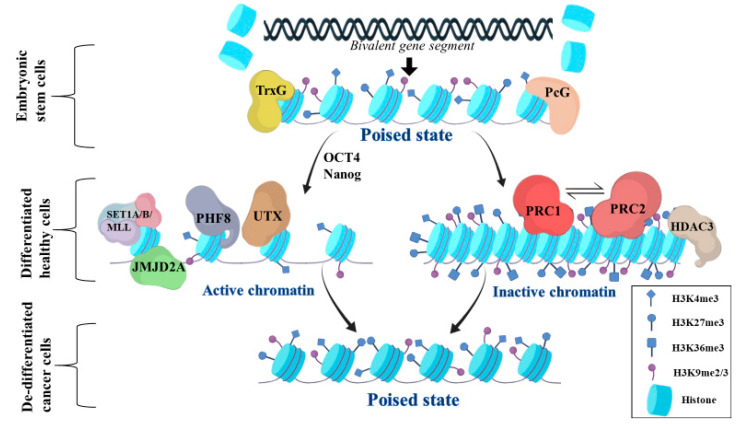
Bivalent genes and cell differentiation. Embryonic stem cells (ESCs) have the potential to quickly change the expression levels of a gene through the mechanism of bivalency. Bivalent genes carry both inducing signals, such as H3K4me3 methylation, as well as repressive signals, such as H3K27me3 methylation, H3K9me2/3 methylation, and H3K36me3 methylation. The formation and control of bivalent genes are mostly regulated by TrxG and PcG that keep bivalent genes in a ‘poised’ state. Activating signals induce the deposition of H3K4me3 by SET1A/B/MLL, the removal of H3K27me3 marks by UTX, and the removal of H3K9me2/3 marks by PHF8 and JMJD2A, making chromatin more active and transcriptionally accessible. On the other hand, inhibitory signals promote the deacetylation of H3K4 by HDAC3 and the deposition of H3K27me3 by PRC2, which aim to suppress gene transcription and induce chromatin inactivation. PRC1 deposits the H3K36me3 mark, which interacts with PRC2 and promotes its binding. PRC1 can also be recruited by PRC2. Previously differentiated cells that undergo malignant transformation may acquire a stem cell-like phenotype by silencing the expression of some bivalent genes while maintaining the expression of others. This leads to the dedifferentiation of neoplastic cells and promotes the regeneration of stem cell properties in cancer cells.

The bivalent chromatin state is defined by the co-occurrence of activating and repressive histone epigenetic modifications in the same DNA segment, which work in concert to enhance or silence gene expression. In other words, it is characterized by the acquisition and maintenance of both repressing and activating histone marks at the same time during the cell cycle, depending on the received signals (Figure 1). More specifically, during early development, the pluripotent embryonic stem cells (ESC) are multiplied and undergo differentiation in specific time-points, controlled with tremendous accuracy to generate a fully developed organism. To carry out this task, ESCs need to rapidly change the transcriptional status of their genes in response to external stimuli to keep up with the tight developmental schedule. An ingenious way of achieving this rapid change in gene expression is conferred by bivalency. Bivalent genes usually code for crucial transcription factors [20], controlling complex developmental procedures and, most importantly, cell differentiation [21]. Among the genes that fall in this category are the HOX genes, a subset of homeobox genes with established effects on early embryonic development [22], as well as Spindlin1. Spindlin1 acts as a histone reader protein [23], a regulator of gene transcription in early development [24], and participates in chromosomal segregation during cell division [25]. Other genes include bone morphogenic protein 2 (*BMP2*) and bone morphogenic protein 4 (*BMP4*), c-*MYC*, *MET* Proto-Oncogene, Receptor Tyrosine Kinase (*MET*), *MYB* Proto-Oncogene, Transcription Factor (*MYB*), SRY-Box Transcription Factor 9 (*SOX9*), Twist Family BHLiH Transcription Factor 1 (*TWIST1*) and *EGFR* [26,27]. Overall, bivalent genes provide cells with a quick way of altering their expression profiles in order to respond to differentiation signals.

The two most common histone marks that are present at the transcription start site of the bivalent gene promoters are the activating H3K4me3 and the repressive H3K27me3 [28,29]. The H3K4me3 mark is restored before the beginning of mitosis, whereas the repressive H3K27me3 mark is deposited after mitosis [18]. Other histone modifications that have also been detected in bivalent chromatin domains include the combination of H3K4me3-H3K9me3/2 and the H3K36me3-H3K27me3 marks [30,31]. Of importance, histone methylation depends on DNA methylation, and this crosstalk is achieved through interactions between histone and DNA methyltransferases. More specifically, the presence of methyl groups on a DNA segment can affect histone modifications in the overlying nucleosomes and vice versa, through the action of methyl binding proteins. Histone methylation can cause readily reversible local heterochromatin formation, whereas DNA methylation allows for stable long-term heterochromatin establishment throughout many cell divisions. Therefore, DNA methylation, with its autonomous maintenance mechanism, can drive histone methylation of unmethylated DNA regions in subsequent divisions by recruiting the necessary enzyme machinery [32].

## 3. Regulation of Bivalent Genes Promoters and Enhancers

The formation and maintenance of the bivalent regions are mainly regulated by the controlled action of proteins Polycomb (PcG) and Trithorax (TrxG) [20], which is achieved by the regulation of their access to target genomic loci. The CpG islands, commonly found at vertebrate gene promoters, are tightly correlated with the bivalent state [33]. The CpG islands of bivalent genes are mainly primed with the H3K4me3 mark [21]. It has become apparent that in the context of bivalent gene transcription, there is a balance between repressive signals which promote H3K27me3 mark deposition and activating signals which tip the scale towards the deposition of the H3K4me3 mark, thus promoting transcription [21].

### 3.1. Activation of Bivalent Gene Promoters

In regard to the activation of bivalent genes, SET Domain Containing 1A, Histone Lysine Methyltransferase/SET Domain Containing 1B with Histone Lysine Methyltransferase/Histone-Lysine N-Methyltransferase 2A (*SET1A*/*B*/*MLL*) complexes are responsible for the deposition of most H3K4me3 marks. Their recruitment is mediated either by the CXXC-Type Zinc Finger Protein (CXXC) domain-containing proteins or by the ten-eleven translocation (TET) enzymes, O-Linked N-Acetylglucosamine (GlcNAc) Transferase (OGT), and histone variants [34].

When a signal activates the transcription factor octamer-binding transcription factor 4 (OCT4) or Nanog Homeobox (Nanog), the deposition of the H3K4me3 mark is enhanced. Elevated H3K4me3 deposition along with transcription factor binding can displace the inhibitory signals (e.g., PcG proteins) either through competition for the binding site or through repulsion of the Polycomb repressive complex 2 (PRC2) (Figure 1). Additionally, H3K4 acetylation can also promote transcription of bivalent genes while Histone Deacetylase 3 (HDAC3), which removes the acetyl groups from H3K4, serves mainly the opposite function [35]. Furthermore, genes receiving these activating signals may also decrease their H3K27me3 load by certain demethylases, such as Lysine Demethylase 6A (UTX), in order to further decrease their inhibitory signals [36]. The activating signal for bivalent genes is tightly regulated and occurs only at certain developmental phases when needed to induce transcription of ‘poised’ genes. Before the activation signal of bivalent gene transcription, the simple presence of H3K4me3, which is not reinforced by the *SET1A*/*B*/*MLL* complexes, is not sufficient to prevent PRC2 from binding and depositing H3K27me3 marks, thus promoting the formation of an asymmetrical nucleosome which minimizes or completely ‘shuts down’ bivalent gene expression [20].

This multi-subunit protein complex PRC2 cooperates with PRC1 to promote their mutual recruitment. PRC1 can recruit PRC2 by increasing the condensation of chromatin, while PRC2 is able to recruit PRC1 using the H3K27me3 mark. The demethylases which decrease the H3K27me3 load are still able to act at this phase, promoting the balance between H3K4me3 and H3K27me3 [37]. Under this balance, the bivalent genes remain silenced or minimally expressed in the undifferentiated cells, and they are ready to be activated when the appropriate differentiation signal is received [34]. As observed in hematopoietic stem cells, bivalent gene histone modifications regulate the active pool of stem cells towards their renewal or differentiation, giving rise to different mature hematopoietic cell types [34]. Therefore, ESCs do not express HOX genes since all HOX clusters are fully modified by H3K27me3. However, at their promoter regions, this epigenetic pattern co-exists with H3K4me3, constituting a bivalent region and keeps these genes poised for potential future expression during differentiation, where a progressive loss of H3K27me3 is achieved followed by a concurrent gain of H3K4me3. Again, the PcG and TrxG groups of proteins regulate H3K27me3 and H3K4me3 deposition, respectively, and are important in the epigenetic regulation of HOX genes [20]. Finally, the H3K4me3-H3K9me3/2 marks have also been demonstrated to exhibit similar effects to H3K4me3-H3K27me3 in the expression of genes crucial to differentiation in trophoblast stem cells, extraembryonic endoderm stem cells, and preadipocytes [38]. At the H3K4me3-H3K9me3/2 balance, the PHD Finger Protein 8 (PHF8) histone demethylase can further promote gene transcription signaling via its Jumonji (JmjC) and PHD domains. The PHD domain acts to promote H3K9me2/1 demethylation by inducing the JmjC domain, thus enhancing gene transcription. Jumonji Domain Containing 2A (JMJD2A), another H3K9me3 demethylase that can recognize H3K4me3 marks, may reduce the H3K9me3 load on histones and promote transcription [39].

### 3.2. Suppression of Bivalent Gene Promoters

On the other hand, H3K36me3-H3K27me3 marks have been shown to contribute to the inhibition of bivalent gene transcription. The study of Cai et al. [31] demonstrated that the H3K36me3 mark is crucial for avidly anchoring PRC2 in the polycomb-like (PCL) family of Tudor motifs in order to catalyze H3K27me3 deposition in the already H3K27me3 positive promoters or nucleosomes. This shows that the H3K36me3 mark is associated with an inhibitory effect on bivalent gene expression and could serve as a recognition mark for H3K27me3 deposition [31].

### 3.3. Regulation of Gene Enhancers

Concerning the epigenetic histone modifications of gene enhancers, it has been shown that these regulatory elements are tightly correlated with increased levels of the H3K4me1 mark [40]. Although this mark is not specific for gene enhancers, it was significantly elevated in gene enhancers compared to gene promoters. Furthermore, active gene enhancers were also enriched with the H3K4me2/3, H3K9ac, as well as the H3K27ac marks [41]. In this context, a subset of enhancers was observed, which carried only the H3K4me1 mark. These enhancers are “poised” and linked to genes crucial for the early steps of embryogenesis, which were inactivated in embryonic stem cells [41].

Despite the technical difficulties in the precise localization of the above-mentioned bivalent regions, recent studies have identified several bivalent genes in non-pluripotent cells that are different from those found in ESC [20]. Emerging data demonstrate that bivalency plays an important role in the dedifferentiation of cancer cells and is discussed in the following section. It has been suggested that a part of the ESC bivalency becomes re-established in some cancer types, a phenomenon known as “oncofetal epigenetic control” [34].

## 4. Role of Bivalent Genes in Cancer

Current data point towards the crucial role of bivalent genes and their regulation in different cancer cell types. Several genes in tumor cells have been demonstrated to possess both the H3K9me3/2 and H3K27me3 repressive marks in their promoters, as well as decreased levels of H3K4me3 and H3K9 acetylation, which are normally involved in increased transcriptional activity [42].

In this context, cancer treatment with demethylating agents such as 5-aza-2′-deoxycytidine or knockout of *DNMT1* in colon cancer has led to a similar balance state that is found in ESCs. These results show that by using the demethylating agents or knocking out the *DNMT1* gene, it was possible to restore bivalency in the genes that were previously repressed. In these cells, the repressive H3K27me3 mark levels were retained, while the H3K4me3 marks were increased [43]. This leads to the conclusion that cancer cells were able to exploit bivalency in order to suppress the expression of genes related to differentiation, senescence, or apoptosis when necessary to enhance their growth [44].

At the same time, abnormal gene silencing has also been studied in cancer with respect to PcG proteins and their complexes. Almost half of the genes which carry CpG island hypermethylation in colon cancer were shown to be genes that in ESC and embryonic progenitor cells carry the PcG mark and exhibit bivalency [45,46]. Furthermore, these genes can interact with DNMTs to induce the repression of selected genes in adult cancer [47]. Moreover, the Enhancer of Zeste 2 Polycomb Repressive Complex 2 Subunit (EZH2), the PcG protein in the PRC2/3 which catalyzes H3K27me3 trimethylation, has been overexpressed in cancer and was associated with tumor progression and prognosis in prostate, breast, melanoma, endometrium, and bladder and gastric cancers [48,49,50,51]. EZH2 has been linked to abnormally silenced genes via hypermethylation and depletion of EZH-induced cancer cell growth arrest in multiple myeloma [52]. In a similar way, the Chromobox protein homolog 7 (CBX7) was shown to induce methylation of gene promoters and suppress gene expression in adult cancer [47], confirming the implication of histone modifications and bivalent genes in tumorigenesis.

## 5. Bivalent Genes Regulate Tumor Phenotype in Gliomas

A total number of 1.435 frequently bivalent regions have been detected in primary GBMs, covering 1.511 individual genes [53]. It has been demonstrated that transcriptionally active genes in gliomas such as calmodulin (*CALM1*) are characterized by active histone marks, whereas transcriptionally silent genes, such as keratin 72 (*KRT72*), display repressive histone modifications [53].

Bivalent domains were marked by the punctate H3K4me3 and exhibited a broader distribution of H3K27me3. It was demonstrated that bivalent genes were minimally expressed but were less methylated than the polycomb and heterochromatin silenced regions [53]. Bivalent gene transcription was thus slightly greater than that of the polycomb-silenced state but lower than the gene transcription near active promoters or enhancers. Additionally, the multifunctional binding sites for the CCCTC-binding factor (CTCF), which is a transcription regulator and a core architectural protein participating in the three-dimensional organization of the genome [54], were characterized by high levels of methylation. Areas with a specific mark have the tendency to get clustered together, and therefore active marks, repressive marks, and CTCF binding sites can form distinct groups [53].

**Figure 2:**
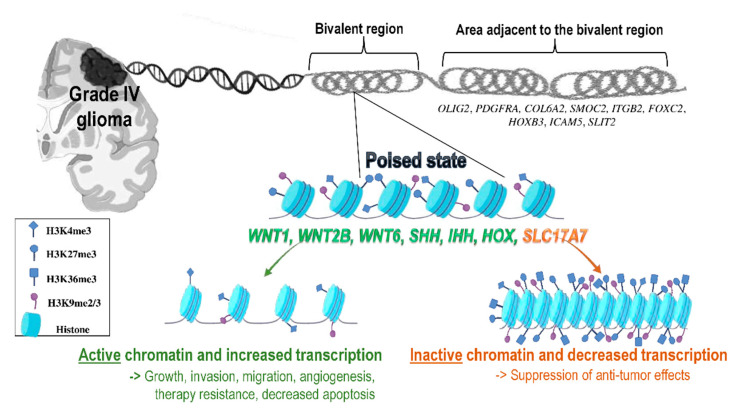
Bivalent regions and associated genes in gliomas. Several bivalent genes have been detected in glioblastomas, being nearly always silent and poised for subsequent activation during tumor progression. Upon activation, these genes are associated with increased growth, invasion, migration, angiogenesis, therapy resistance, and decreased apoptosis. At the same time, tumor suppressor genes can also be bivalently controlled, including *SLC17A7*, which is downregulated in gliomas. Moreover, several genes that characterize GSCs have been detected adjacent to bivalent regions, including *OLIG2*, which is required for the proliferation of GSCs and *PDGFRA*, *COL6A2*, *SMOC2*, *ITGB2*, *FOXC2*, *HOXB3* regions that are associated with angiogenesis, cellular migration, and invasive growth. Additionally, a few cancer-protective genes, such as *ICAM5* and *SLIT2,* which are associated with better survival of glioma patients, are also present in these regions.

Among the genes with bivalent regions in GBMs detected by Hall et al., 840 genes demonstrated high enrichment for pattern specification, Wnt signaling, embryonic development and transcription factor DNA-binding domains [53]. Bivalent regions were also extensively interconnected and formed a network, mainly composed of Wnt (WNT1, WNT2B, WNT6), and Hedgehog (SHH, IHH) signaling associated transcription factors as well as HOX genes, with *IHH*, *SHH*, and *WNT1* being nearly always silent, but poised for subsequent activation as differentiation progresses (Figure 2). Wnt and Hedgehog signaling pathways regulate epithelial-to-mesenchymal transition (EMT), invasion, and proliferation in cancer cells. In respect to genes within the HOXB domain, these are often bivalent in Proneural (PN) tumors, but they are normally expressed in Mesenchymal (MES)/Classical (CL) tumors.

Concerning the implication of HOX gene family in gliomas, previous studies have reported that the increased expression of nine genes (*HOXA6*, *A7*, *A9*, *A13*, *B13*, *D4*, *D9*, *D10*, and *D13*) may contribute to their malignant behavior, growth, and invasion [22], cell viability, migration, angiogenesis, stem-cell capacity, therapy resistance and decreased cell death/apoptosis in adult glioma cells [55]. Deregulation of HOX-D genes may also participate in the pathogenesis of low-grade pediatric gliomas [56]. Of importance, HOX genes and their transcription factors were the most enriched among the bivalent genes studied, suggesting a functional connection between these bivalent regions and glioblastoma tumorigenesis. This is possibly mediated through a Hedgehog and Wnt-mediated transcriptional response, capable of driving the production of multipotent stem cells (SCs) from more differentiated bulk tumor cells [53].

As mentioned above, epigenetic marks, mostly polycomb-associated in ESCs, are under strict control at HOX loci and regulatory regions in order to ensure the correct tuning between HOX activation and repression, which is an example of the crucial epigenetic plasticity needed for homeostatic development [20]. This evidence possibly signifies that even though Glioma Stem Cells (GSCs) comprise a small part of the bulk tumor, the gene bivalency observed in tumors may contribute to the dedifferentiation of mature cancer cells towards a more stem cell-like and plastic phenotype since this ability to switch between the active or repressed state in bivalent regions is a hallmark of ESCs that is normally lost upon differentiation.

This theory is further supported by the fact that many genes expected to be GSC-specific were also highly expressed in the studied tumors, indicating that the genes regulating GSC programming are also present in any glioma cell. Recent studies conducted in cultured GSCs have similarly described that stemness in GBMs is partly controlled by HOX gene epigenetic regulation [57], as well as Wnt signaling. In this regard, polycomb repression is thought to activate Wnt, which then feeds back upon loci, encoding highly active transcription factors in GSCs [58].

Genes adjacent to commonly bivalent regions include genes of GSCs, such as the Oligodendrocyte Transcription Factor 2 (*OLIG2*), required for the proliferation of GSCs and Platelet Derived Growth Factor Receptor Alpha (*PDGFRA*), as well as several genes active in MES/CL tumors, such as Collagen Type VI Alpha 2 Chain (*COL6A2*), SPARC Related Modular Calcium Binding 2 (*SMOC2*), Integrin Subunit Beta 2 (*ITGB2*), Forkhead Box C2 (*FOXC2*), and *HOXB3*, which are associated with angiogenesis, cellular migration, and invasive growth. However, bivalent regions are also adjacent to active genes which are cancer-protective in PN tumors, such as Intercellular Adhesion Molecule 5 (*ICAM5*), an intracellular adhesion molecule, regulating interactions between neurons and microglia, Slit Guidance Ligand 2 (*SLIT2*), which guides the axons of the developing forebrain and is associated with better survival in glioma patients [53].

Interestingly, a recent study also demonstrated that the tumor suppressor gene Solute Carrier Family 17 Member 7 (*SLC17A7*) was also bivalently controlled in GBM [59]. Downregulation of both mRNA and protein levels of SLC17A7 was observed in tumor cells compared to normal brain tissues, and it was suggested that cancer cells take advantage of bivalency to silence this gene, preventing its tumor-suppressive activity (Figure 2). Indeed, overexpression of this gene resulted in reduced proliferation, migration, and invasion in GBM cells.

Moreover, several enhancers defined by co-localization of both H3K27ac and H3K4me1 were previously detected in medulloblastoma and in the U87MG glioma cell line. Notably, in GBM, enhancers were found to be enriched for 5-hydroxymethylation (5hmC), in comparison to promoters, bivalent regions, polycomb silenced regions, and background genome 5hmC levels [53]. Some of the genes associated with these enhancers have been detected upregulated in MES/CL tumors and promote cellular invasion and angiogenesis, which is a hallmark of mesenchymal GSCs [60,61]. Examples of these genes include the Podocalyxin-like (*PODXL*), which favors cell migration, and Matrix metalloprotease 11 (*MMP11*), which cleaves the extracellular membrane, induces tumorigenesis and cellular invasion [62]. Additional genes include the Ca^2+^ binding protein, S100A16, which participates in the EMT in breast cancer [63], the protein kinase FAM20C that promotes proliferation in triple-negative breast cancer, being also a marker of mesenchymal GSCs [64] as well as the LIM Domain Only 2 (*LMO2*) which is a pro-angiogenic gene [65] and a GSC marker [61]. On the other hand, other enhancer-associated genes are known to be upregulated in PN tumors and are associated with increased survival and a less aggressive phenotype, such as AKT Serine/Threonine Kinase 3 (*AKT3*) and dynamin-1 (*DNM1*), as well as Neural cell adhesion molecule 1 (*NCAM1*), which disfavors invasion [66].

### DNA Methylation Confers an Additional Control of Bivalent Gene Expression in Gliomas

It has been proposed that bivalency predisposes genes to hypermethylation and to global transcriptional changes in tumor cells. These changes mainly occur in genes with DNA methylation-associated defects, independently of their association with gain or loss of expression and less often in genes without DNA methylation defects [67]. DNA hypermethylation may occur due to enhanced activity of two DNA methyltransferases (*DNMT1* and *DNMT3b*), surrounding bivalent promoters in cancer cell lines [68]. In this way, genes with bivalently modified chromatin are more sensitive to deregulation in IDH-wild type gliomas, and the dysregulation of H3K27me3 dynamics in bivalent regions accounts for the transcriptional alterations in glioma cells [67].

In parallel, it was demonstrated that the loss of bivalency results in the aberrant expression of developmental tumor genes, such as HOX genes. The combination of these findings led to the proposal that the malfunction of transcriptional readiness in bivalent areas is the common denominator of hypermethylation, loss of bivalency and finally increased gene expression. In more detail, deregulation of silenced genes might cause a subsequent genome-wide activation of previously poised polymerases, inducing a transient gene expression. This incident may sometimes cause adequate promoter DNA methylation, loss of activating chromatin patterns, and gradual re-silencing of genes. But this is not always the case since other genes may recruit alternative promoters or remain active for unknown reasons, which ultimately leads to a mixed-signal of both DNA hypermethylation, as well as upregulated gene expression in tumors [68].

Overall, the concept of bivalency and its association with gene expression alterations forms the basis of plasticity, stemness, and heterogeneity in gliomas. As mentioned above, loss of its tight regulation predisposes tumor cell genes to hypermethylation, alters their expression, and contributes to cancer onset by favoring the expression of stemness -associated genes. Importantly, it needs to be pointed out that the number of bivalent genes and their expression products differs not only between different cancer types but even within the same cancer subtypes. This variability reflects the heterogeneous nature of tumor cell populations, in which bivalency plays an important role [34] by affecting gene expression. Exploring novel bivalent tumor genes and specific targeting of the above-mentioned epigenetic interactions is expected to offer new therapeutic opportunities for the management of gliomas in the future.

## 6. Targeting Options of Bivalent Genes

Bivalency could be targeted therapeutically in order to suppress tumor-promoting genes and also re-express tumor suppressor genes, such as *SLC17A7* [59], mentioned above. It could also be explored as an adjunct therapeutic scheme to current chemotherapy regimens with the aim to increase tumor cell resistance.

In this context, it is important to note that the expression of some HOXA cluster genes (*HOXA9* and *HOXA10*) has been associated with lower sensitivity to the chemotherapeutic drug temozolomide (TMZ) in glioma patients. Specifically, *HOXA5* expression was demonstrated to decrease the sensitivity of tumor cells to radiotherapy both in vitro and in vivo [55]. The tumorigenic properties of these genes have suggested the use of small-molecule modulators in gliomas in combination with agents that affect the Wnt and Hedgehog signaling pathways as a promising approach for exploring more effective cancer subtype-specific therapies [53].

Previous treatment approaches involve mainly targeting mutations of epigenetic enzymes, such as gain-of-function mutations in DNA methylases, as well as other histone modifiers and readers. Their aim was to alter DNA methylation and histone modifications to epigenetically modify tumor phenotypes. Such examples include the Lysine-Specific Histone Demethylase 1A (LSD1) inhibitors, histone methyltransferase EZH2 inhibitors, Protein arginine methyltransferase 5 (PRMT5) inhibitors, Disruptor of telomeric silencing 1-like (DOT1L) inhibitors, as well as Bromodomain and extraterminal (BET) protein inhibitors [34]. The Deazaneplanocin A (DZNep), an EZH2 inhibitor, was tested along with the small-molecule compound AC1Q3QWB (AQB) in vitro, as well as in orthotopic breast cancer and GBM patient-derived xenograft (PDX) models, and was demonstrated to significantly reduce tumor growth [69]. Moreover, the small-molecule inhibitors of EZH2, Tazemetostat, and CPI-1205 are currently tested in clinical trials, targeting the overexpression of EZH2, observed in several cancer types. In a preclinical study of pediatric rhabdomyosarcomas, EZH2 inhibition was shown to promote cell differentiation and decrease aggressiveness [70], inspiring several clinical studies to be conducted in different types of tumors [71,72].

Specific targeting of Histone deacetylases (HDACs) has also been proposed as a therapeutic modality in both pediatric and adult gliomas. Although most HDAC inhibitors exert little to no effect when used alone [73], their combination with chemotherapy and radiotherapy showed some encouraging results, which are currently under investigation. Among the HDAC inhibitors used, valproic acid was well-tolerated with promising results, especially in adults. Median survival was increased by a great margin [74], compared to radiation response-modifying drugs used in the treatment combination for GBM, such as erlotinib, enzastaurin, and poly-ICLC [75,76,77]. Furthermore, valproic acid was more effective compared to other antiepileptic drugs in increasing patients’ survival [78]. Other HDACs under investigation include Vorinostat, Panobinostat, and Romidepsin [79,80]. The study of Grasso et al. [81] demonstrated that treatment with the pan-HDAC inhibitor, Panobinostat in combination with the histone demethylase inhibitor GSKJ4 increased H3 acetylation and H3K27 methylation, reversing partially the H3.3 K27M global hypomethylation phenotype in diffuse intrinsic pontine gliomas (DIPG) bearing the H3.3 K27M mutation. The combination of Panobinostat with GSKJ4 further decreased cell viability, highlighting the need for further clinical research on HDAC inhibitors.

A more understudied therapeutic strategy includes targeting epigenetic abnormalities along with co-existent gene mutations to treat gliomas. Tayler et al. [82] demonstrated that 21% of pediatric DIPG are heterozygous for the mutated Activin A Receptor Type 1 (ACVR1) gene, which is also observed in patients with the connective tissue disorder Fibrodysplasia ossificans progressive (FOP). The product of this gene is the activin A type I receptor serine/threonine kinase ALK2. When mutated, it promotes the activation of the transforming growth factor-β pathway, which is dependent on bone morphogenic protein (BMP). ACVR1 mutations were correlated with histone H3.1 K27M mutated-DIPG, raising interest towards BMP targeting to evaluate its role in the development of DIPG [82].

In another study by Hashizume et al. [83], inhibition of the H3K27 demethylase, Jumonji Domain Containing 3, Histone Lysine Demethylase (JMJD3) in pediatric high-grade gliomas (HGG) using GSKJ4 exhibited a 50% decrease in growth, increased apoptosis, and inhibition of clonal growth of H3.3 K27M glioma cells in vitro. JMJD3 depleted glioma cells showed no appreciable reduction in growth after treatment with GSKJ4 [84]. The in vivo part of their study was conducted on athymic mice with brainstem K27M glioma xenografts, in which a significant reduction in tumor growth was observed, along with extended survival.

Finally, in a more wide-range retrospective cohort study was demonstrated that patients with low levels of H3K4 methylation were correlated to worse prognosis, while low levels of H3K18 acetylation were associated with a more favorable survival [85]. These data further suggest the use of HDAC inhibitors as treatment options, and the evaluation of histone modifications, as prognostic markers and predictors of chemotherapy response.

All these examples also highlight the therapeutic potential of targeting the enzymes that mediate bivalency [34]. Gene expression, however, also involves additional levels of regulation, such as miRNAs presence, which may also need to be taken under consideration for effective therapeutic targeting.

## 7. Conclusions—Future Perspectives

In conclusion, emerging data point towards the important role of epigenetic regulation of bivalent genes in cancer, opening a new direction in the development of novel therapeutic approaches for gliomas. Bivalent genes offer cancer cells the advantage of plasticity through their ability to be effectively and easily regulated by epigenetic alterations according to different signals. This provides neoplastic cells with the option of turning their genes “on” or “off” in order to acquire a tumorigenic phenotype based on the concept of stemness. In more detail, in this context of plasticity, bivalent gene regulation of developmental genes allows for the expression of stem cell genes, such as those of the *HOX* family. Moreover, each cancer type is comprised of a heterogeneous population of cells with unique alterations in different bivalent genes, which occur via a complex network of epigenetic alterations that could be therapeutically targeted.

However, several issues need to be addressed before epigenetic targeting can be applied clinically, including complete transcriptional profiling of genes controlled by enzyme modulators and revelation of the associated changes upon treatment. Additionally, the control and maintenance of bivalency are achieved through the interplay between different histone modifications, enzymes, and cofactors, stressing the level of complexity that needs to be considered before selecting a single enzyme inhibitor, without clear knowledge of its effects on the rest of the epigenetic machinery. It is important to note that bivalent genes comprise only a small part of gene regulation, which also involves DNA modifications apart of histones, post-transcriptional and post-translational changes, as well as other potential genomic targets, such as miRNAs, lncRNAs, which may further affect the outcome of histone modification targeting.

Moreover, it is necessary to identify the different bivalent genes that are present in each cancer type and subtype since this variability reflects the heterogeneous nature of tumor cell populations and may lead to differential outcomes of therapeutic targeting. In this context, the lack of specificity of some epigenetic drugs/agents needs to be pointed out. For instance, drugs like DZNep are global histone methylation inhibitors and not H3K27-specific [55]. The elucidation of the epigenetic signatures of each cancer patient may thus be necessary before using targeted therapies to avoid adverse effects that may stem from the differential epigenetic changes among individuals.

Finally, due to the intricate nature of bivalency, its exact role in tumor formation and progression has yet to be explored. Additional studies are necessary to determine whether bivalent genes are the determinants of a specific cancer subtype or whether bivalent genes are the consequence of malignant transformation and how they can specifically influence tumor cell fate.

Although several issues need to be clarified before applying these novel therapeutic strategies, there is no doubt that gene bivalency is a key influencer of tumor progression that determines cancer heterogeneity and plasticity. Future elucidation of the bivalent genes and underlying mechanisms offers new opportunities in identifying novel biomarkers for diagnosis and disease progression, as well as in developing patient-specific and selective therapeutic approaches on the basis of personalized and precision medicine.

## Data Availability

Not applicable.

## Abbreviations

TrxG	Trithorax
PcG	Polycomb
SET1A/B/MLL	SET Domain Containing 1A, Histone Lysine Methyltransferase/SET Domain Containing 1B
PHF8	PHD Finger Protein 8
JMJD2A	Jumonji Domain Containing 2A
UTX	Lysine Demethylase 6A
OCT4	Octamer-binding transcription factor 4
Nanog	Nanog Homeobox
PRC2	Polycomb repressive complex 2
PRC1	Polycomb repressive complex 1
HDAC3	Histone deacetylase 3
OLIG2	Oligodendrocyte Transcription Factor 2
PDGFRA	Platelet Derived Growth Factor Receptor Alpha
COL6A2	Collagen Type VI Alpha 2 Chain
SMOC2	SPARC-related modular calcium-binding protein 2
ITGB2	Integrin Subunit Beta 2
Forkhead Box C2	Forkhead Box C2
HOXB3	Homeobox B3
ICAM5	Intercellular Adhesion Molecule 5
SLIT2	Slit Guidance Ligand 2
WNT1	Wnt Family Member 1
WNT2B	Wnt Family Member 2B
WNT6	Wnt Family Member 6
SHH	Sonic Hedgehog
IHH	Indian hedgehog homolog,
HOX	Homeobox
SLC17A7	Solute Carrier Family 17 Member 7
H3K4me3	Histone 3 Lysine 4 trimethylation
H3K27me3	Histone 3 Lysine 27 trimethylation
H3K36me3	Histone 3 Lysine 36 trimethylation
H3K9me2/3	Histone 3 Lysine 9 di- or trimethylation
